# Bispectral Analysis of Heart Rate Variability to Characterize and Help Diagnose Pediatric Sleep Apnea

**DOI:** 10.3390/e23081016

**Published:** 2021-08-06

**Authors:** Adrián Martín-Montero, Gonzalo C. Gutiérrez-Tobal, David Gozal, Verónica Barroso-García, Daniel Álvarez, Félix del Campo, Leila Kheirandish-Gozal, Roberto Hornero

**Affiliations:** 1 Biomedical Engineering Group, University of Valladolid, 47002 Valladolid, Spain; gonzalo.gutierrez@gib.tel.uva.es (G.C.G.-T.); veronica.barroso@gib.tel.uva.es (V.B.-G.); dalvarezgo@saludcastillayleon.es (D.Á.); fsas@telefonica.net (F.d.C.); robhor@tel.uva.es (R.H.); 2 CIBER-BBN, Centro de Investigación Biomédica en Red en Bioingeniería, Biomateriales y Nanomedicina, 28029 Madrid, Spain; 3 Department of Child Health and the Child Health Research Institute, The University of Missouri School of Medicine, Columbia, MO 65212, USA; gozald@health.missouri.edu (D.G.); gozall@health.missouri.edu (L.K.-G.); 4 Sleep-Ventilation Unit, Pneumology Service, Río Hortega University Hospital, 47012 Valladolid, Spain

**Keywords:** pediatrics, obstructive sleep apnea, heart rate variability, bispectral analysis, multi-layer perceptron neural network

## Abstract

Pediatric obstructive sleep apnea (OSA) is a breathing disorder that alters heart rate variability (HRV) dynamics during sleep. HRV in children is commonly assessed through conventional spectral analysis. However, bispectral analysis provides both linearity and stationarity information and has not been applied to the assessment of HRV in pediatric OSA. Here, this work aimed to assess HRV using bispectral analysis in children with OSA for signal characterization and diagnostic purposes in two large pediatric databases (0–13 years). The first database (training set) was composed of 981 overnight ECG recordings obtained during polysomnography. The second database (test set) was a subset of the Childhood Adenotonsillectomy Trial database (757 children). We characterized three bispectral regions based on the classic HRV frequency ranges (very low frequency: 0–0.04 Hz; low frequency: 0.04–0.15 Hz; and high frequency: 0.15–0.40 Hz), as well as three OSA-specific frequency ranges obtained in recent studies (BW1: 0.001–0.005 Hz; BW2: 0.028–0.074 Hz; BWRes: a subject-adaptive respiratory region). In each region, up to 14 bispectral features were computed. The fast correlation-based filter was applied to the features obtained from the classic and OSA-specific regions, showing complementary information regarding OSA alterations in HRV. This information was then used to train multi-layer perceptron (MLP) neural networks aimed at automatically detecting pediatric OSA using three clinically defined severity classifiers. Both classic and OSA-specific MLP models showed high and similar accuracy (Acc) and areas under the receiver operating characteristic curve (AUCs) for moderate (classic regions: Acc = 81.0%, AUC = 0.774; OSA-specific regions: Acc = 81.0%, AUC = 0.791) and severe (classic regions: Acc = 91.7%, AUC = 0.847; OSA-specific regions: Acc = 89.3%, AUC = 0.841) OSA levels. Thus, the current findings highlight the usefulness of bispectral analysis on HRV to characterize and diagnose pediatric OSA.

## 1. Introduction

Obstructive sleep apnea (OSA) is a common respiratory disorder affecting up to 5% of the general pediatric population [1]. OSA is characterized by the occurrence of either total upper airway obstruction (apnea events) and/or events of significant airflow reduction (hypopnea events) during sleep, leading to decreased blood oxygen saturation and/or sleep fragmentation [2,3]. As a result, pediatric OSA has been associated with several adverse cognitive and cardiovascular consequences, such as learning deficits and reduced academic performance [4,5], as well as alterations in autonomic regulation and vasomotor tone, increases in systemic and pulmonary vascular blood pressure, nocturnal cardiac strain and both left and right ventricular hypertrophy [1,3]. The increased cognitive and cardiovascular risks obviously threaten the long-term cardiovascular health [1,3,6] and academic potential of children [7], such that early detection and treatment of pediatric OSA are essential.

Nocturnal laboratory-based polysomnography (PSG) is considered the standard diagnosis technique for pediatric OSA. This test not only allows for the detection of the presence or absence of pediatric OSA but also enables estimates of OSA severity [8,9]. During a PSG, a set of leads is placed on the child’s body to register biological signals, including an electrocardiogram (ECG), electroencephalography, oximetry (SpO_2_) or airflow, among others [8]. Then, medical experts evaluate these signals following well-established rules to extract indices of respiratory disturbance [10]. Among those indices, the common choice to illustrate and report OSA severity is the Apnea–Hypopnea Index (AHI), which reflects the number of total apneic and hypopneic events per hour (e/h) of sleep [8,10]. Despite the usefulness of PSG to diagnose pediatric OSA, the procedure needs a specialized laboratory and is resource-intensive and time-consuming. Moreover, the high number of sensors connected to the child along with having to sleep outside of home makes PSG also especially uncomfortable and inconvenient for patients and caretakers [11,12]. These drawbacks have motivated the search for alternatives to diagnose pediatric OSA and to study its consequences while reducing the number of signals required for the diagnosis [11,12,13]. A recently published systematic review [14] analyzed and conducted a meta-analysis on machine learning techniques employed to automatically diagnose pediatric OSA. Among the shortcomings identified in the literature, the authors highlighted that most of the studies were exclusively based on the analysis of SpO_2_ signals. Thus, there exists a lack of studies performing machine learning techniques using other physiological signals as alternative approaches to the gold standard PSG [14].

Evaluation of the adverse cardiovascular implications of pediatric OSA has highlighted autonomic dysfunction as the main reason for cardiac alterations, since the autonomic nervous system (ANS) plays a major role in cardiovascular system regulation [15,16,17]. In this sense, analysis of heart rate variability (HRV) has gained obvious relevance as it reflects the ANS state [17,18]. HRV non-invasively assesses the variations in the heart rate over time, meaning it can be used to analyze cardiovascular modulation due to OSA [18,19,20,21]. As a result, a variety of studies have evaluated HRV in the context of pediatric OSA [16,17,22,23,24,25,26,27,28]. The vast majority of these studies focused on HRV analysis in the temporal or frequency domain. When using these traditional analysis approaches, nonlinearity and non-Gaussianity are ignored [29]. Although HRV signals are usually nonlinear and non-Gaussian in nature [29,30], under some specific conditions, HRV can show dynamics where these nonlinearities are not always present [31]. Nevertheless, in the context of pediatric OSA, the nonlinear dynamics of HRV signals can be increased during sleep [21,32], as well as by cardiac alterations due to OSA [20,21,30,32]. Furthermore, in the present study, the presence of HRV nonlinear dynamics in the pediatric OSA context has been demonstrated (see Appendix C). Despite this, only two of the previous studies on pediatric OSA performed non-lineal analysis of HRV signals using Poincaré plots [17,33]. Notwithstanding, application of bispectral analysis of HRV would likely constitute a further advance, as it allows for reflection of not only nonlinear behaviors but also non-Gaussian and non-stationary events, all the while retaining phase and amplitude information, and being more immune to noise [29]. The unique advantages conferred by the bispectrum analysis properties have led to its application in HRV for some purposes such as evaluation of cardiac state [34,35,36], congestive heart failure [37], major depression [38] or even OSA diagnosis in adults [30].

OSA was initially described in adults, and its cardiac implications and effects on HRV have been extensively studied in recent decades [39,40,41]. Nevertheless, pediatric OSA presents differentiating etiological, diagnostic and treatment considerations [5]. It has led to an increasing interest in the study of pediatric OSA to gain insights into the mechanism underlying pediatric OSA pathogenesis in recent years [3,5]. The advances in the study of pediatric OSA have demonstrated that the long-term effects of the disease in the child population can be avoided with a timely treatment [11]. Accordingly, more stringent rules were defined to diagnose pediatric OSA [10]. In this sense, while an adult is considered as an OSA patient when presenting an AHI = 5 e/h, the presence of an AHI = 1 e/h in children is enough to diagnose pediatric OSA. Similarly, an adult patient with an AHI = 10 e/h is considered as mild OSA, whilst the same AHI in children is considered as severe OSA [42]. Furthermore, an apneic event is scored in adults when it lasts over 10 s, while in children, 6 s is enough to score a respiratory event as apneic [10]. These distinctions between children and adults also produce a marked difference in the HRV alterations due to OSA and consequently affect the HRV bispectrum in a distinct way, evidencing the necessity of independent HRV pediatric study. However, to date, HRV bispectral analysis has never been evaluated in the pediatric OSA context.

Of note, previous bispectral analysis study in adults focused on OSA [30], as well as some of the aforementioned studies [34,35], considered HRV bispectral analysis that was restricted to the non-redundant bispectral region. This region, as a result of the bispectrum symmetric properties, is known to completely define the overall information contained in the bispectrum [29]. Other studies analyzed particular bispectral regions defined based on the classic frequency ranges of HRV analysis [36,37,38]. However, in a study using regular spectral analysis [27], we recently showed that there exist OSA-specific frequency ranges that allow for better characterization of the alterations occurring in HRV in the context of pediatric OSA.

Based on these considerations, we hypothesized that bispectral analysis of HRV contains novel and useful information to characterize and diagnose pediatric OSA. Consequently, the main objectives of this study were to perform, for the first time in the field of pediatric OSA, a characterization and evaluation of bispectral regions bounded with classic and OSA-specific HRV frequency ranges. Therefore, the main novelty of this study is the analysis of HRV bispectral regions defined by classic and OSA-specific frequency ranges to characterize and diagnose pediatric OSA. Furthermore, we propose a novel bispectral parameter, which is analyzed here for the first time.

## 2. Subjects and Signals under Study

The population included in this study was the same as that in a previous work [27] and was composed of 1738 children between 0 and 13 years of age. This cohort consists of two large databases. The first one was generated in the Pediatric Sleep Unit at the University of Chicago (UofC) Medicine Comer Children’s Hospital (Chicago, IL, USA) and involved 981 children referred for a nocturnal laboratory-based PSG due to clinical symptoms suggestive of OSA. The second database was a subset of 757 PSGs performed during the multicenter Childhood Adenotonsillectomy Trial (CHAT) study [43,44].

In regard to the UofC dataset, the Ethics Committee of the UofC Medicine Comer Children’s Hospital approved the protocol (#11-0268-AM017, #09-115-B-AM031 and #IRB14–1241), and the study was conducted in accordance with the Declaration of Helsinki. Additionally, before the acquisition of the ECGs during the PSG, informed consent from all children caretakers was acquired. For comparison purposes, the training–test division carried out was the same as that in our preceding work [27], meaning the 981 children from the UofC sample served as the training set.

Regarding the CHAT set (clinical trial identifier: NCT00560859), information about the rationale, study design and methodological aspects can be perused in the published literature [43,44]. Initially, there were 779 pediatric PSG recordings, but 22 subjects were removed from the study because they did not fulfill the signal pre-processing protocol detailed below. Accordingly, the remaining 757 children were retained as the test set.

The PSG studies from both databases were evaluated by pediatric sleep experts from the different centers who diagnosed the children following the scoring rules established by the American Academy of Sleep Medicine [10]. Subsequently, the AHI was obtained and used to determine OSA severity. In accordance with previous pediatric OSA studies [11,27,42,45,46], we defined four OSA severity groups based on three common AHI cutoff points (1, 5 and 10 e/h). Thus, the severity groups in this study included: no-OSA (AHI < 1 e/h), mild OSA (1 ≤ AHI < 5 e/h), moderate OSA (5 ≤ AHI < 10 e/h) and severe OSA (AHI ≥ 10 e/h) groups. Clinical and demographic data of the children under study are shown in Table 1.

In order to conduct the HRV analysis, ECGs from both datasets were equally pre-processed [27]. First, we removed the initial and last 15 min of the ECG recordings to prevent the signals from periods of early and final artifacts [27]. After this removal, we assessed that, at least, 3 h of sleep recording was available for each child [46,47]. The next step consisted in R peak detection. To carry out this process, the algorithm developed by Benítez et al. [48] was used, which is based on the Hilbert transform and has been evaluated in previous studies [27,49]. Then, HRV signals were derived by measuring the time between consecutive R peaks, i.e., RR intervals [19]. An artifact rejection step was then performed to ensure that all the intervals considered were physiologically plausible (N-N intervals). To this effect, we rejected those RR intervals that did not satisfy the following criteria [21]: (i) RR interval duration was between 0.33 and 1.5 s, and (ii) difference from the previous RR interval was higher than 0.66 s. After the artifact rejection, we confirmed that the duration of signals still surpassed 3 h of sleep. At this point, the remaining HRV samples were not equally distributed among the time; therefore, all the signals were resampled to a constant sampling frequency of 3.41 Hz [21,49], allowing us to perform higher-order spectra (HOS) analyses.

## 3. Methods

The methodology followed can be divided into three stages. First, we conducted a feature extraction stage to characterize each of the bispectral regions considered in the study. Second, we performed an automatic feature selection stage based on the fast correlation-based filter (FCBF) algorithm [50] considering two cases, one for the regions defined by classical HRV frequency ranges, and the other for the regions defined by OSA-related frequency ranges. Finally, a classification stage was conducted based on multi-layer perceptron (MLP) neural networks for the three AHI cutoffs used for partitioning OSA severity groups (1 e/h, 5 e/h and 10 e/h) and for each feature subset resulting from the feature selection stage.

### 3.1. Bispectrum Estimation

The estimation of power spectra has been one of the main tools for the analysis of biological signals for decades [29]. This technique contains information of the autocorrelation sequence, which is enough to characterize Gaussian signals, but information regarding the phase relationship among frequency components, as well as phase coupling, is lost during the process [29,34]. HRV signals, as with many other biological signals, are essentially nonlinear, non-Gaussian and non-stationary [29]. Therefore, power spectrum analysis may not be able to completely characterize changes in HRV series [34]. HOS analysis, meanwhile, contains both amplitude and phase information and can be used to characterize Gaussianity, stationarity and linearity deviations [29].

HOS are spectral representations of higher-order cumulants of a random process [29]. In particular, the bispectrum refers to the HOS for the third-order cumulant, reflecting spectral decomposition of the signal skewness over the frequency [29]. Bispectrum computation is based in the 2D Fourier transform of the third-order cumulant, and it can be defined in terms of the Fourier transform as [29,34]

(1) $$B\left(f_{1},f_{2}\right)=X\left(f_{1}\right)\cdot X\left(f_{2}\right)\cdot X^{*}\left(f_{1}+f_{2}\right),\;\;\;\;\;\;f_{1},\;f_{2}=0,\cdots ,\;f_{N}\;$$

where *X*(*f*) is the Fourier transform of a signal, *f*_1_ and *f*_2_ are the frequency indices, and *f_N_* is the Nyquist frequency. The resultant matrix reflects the phase coupling degree between frequency components for each pair *f*_1_,*f*_2_ [29].

As the bispectrum preserves both amplitude and phase information, it allows for assessment of interactions between signal patterns [29]. Likewise, bispectral analysis is used to evaluate changes in signal Gaussianity, where bispectral values = 0 indicate that signal components are Gaussian, and deviations from the Gaussianity of the components are otherwise detected [29]. Furthermore, bispectral analysis detects linearity deviations through phase coupling between its frequency components [29]. Phase coupling between three harmonic components at the *f*_1_, *f*_2_ and *f*_3_ frequencies and phase angles *φ*_1_, *φ*_2_ and *φ*_3_ is described as *f*_3_ = *f*_1_ + *f*_2_ and *φ*_3_ = *φ*_1_ *+ φ*_2_ [29]. Thus, if phase coupling exists, it means that there are nonlinear interactions between harmonic components [29].

In this work, the HRV bispectrum was estimated employing a Hamming window of 2^10^ samples with 50% overlapping and an FFT of 2^11^ samples. After bispectrum matrix computation, a normalization was applied by dividing each element of the matrix by the sum of all matrix elements as [29,46]

(2) $$B_{N}\left(f_{1},f_{2}\right)=\frac{B\left(f_{1},f_{2}\right)}{BP},\;\;\;\;\;\;f_{1},\;f_{2}=0,\cdots ,\;f_{N}$$

where *BP* is the total bispectral power, measured as the sum of all magnitudes of the bispectral matrix. This normalization was applied in order to ensure that all elements of the matrix were bounded between 0 and 1, reducing subject inter-variability due to physiological conditions other than OSA [46].

### 3.2. Determination of Bispectral Regions

The bispectral matrix, by definition, presents symmetric properties that render evaluation of a triangular non-redundant area sufficient for a full bispectrum characterization [29,34]. This region is commonly named the region of interest (ROI) and satisfies 0 ≤ *f*_1_ ≤ *f*_2_ ≤ *f*_1_ *+ f*_2_ ≤ *f_N_* [29,34]. As it was mentioned in the Introduction section, previous studies analyzing HRV bispectra focused their analysis along the whole ROI [30,34,35]. The analysis of HRV in the frequency domain, however, is commonly performed along the classic HRV spectral bands, i.e., the very low frequency (VLF, 0–0.04 Hz), low frequency (LF, 0.04–0.15 Hz) and high frequency (HF, 0.15–0.4 Hz) bands [18]. Past studies defined sub-band regions inside the bispectral ROI, bound by those frequencies [36,37,38], which we have termed classic bispectral regions. Furthermore, in a previous study applying a common spectral analysis, three pediatric OSA-related spectral ranges for HRV analysis were identified, which outperformed the classic spectral bands for pediatric OSA characterization and diagnosis [27]: BW1 (0.001–0.005 Hz), BW2: (0.028–0.074 Hz) and BWRes (0.04 Hz around HF peak). A detailed explanation of the process that led us to obtain those frequency ranges can be found in Appendix A. Following a similar reasoning to those studies that analyzed classic bispectral regions, three OSA-specific bispectral regions can be defined as bound by those OSA-related frequency ranges.

**Figure 1:**
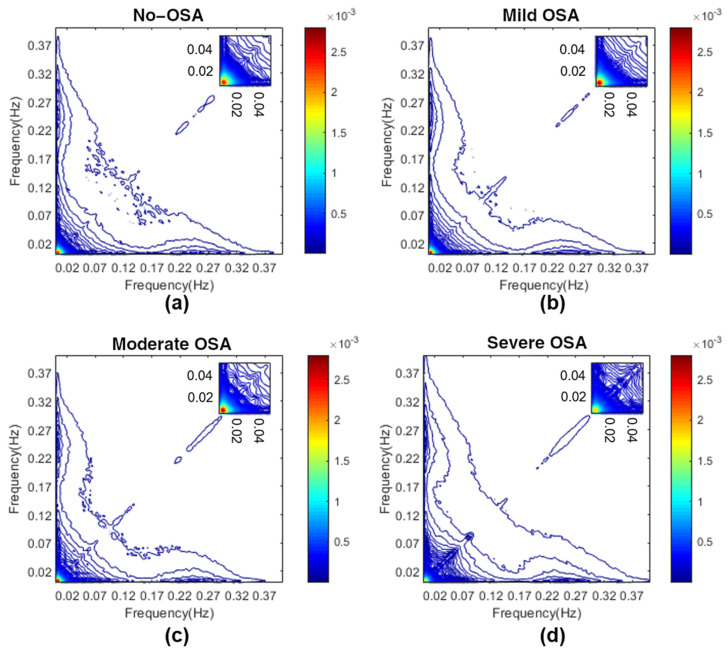
Averaged bispectrum magnitude in the range 0–0.4 Hz in the training set for the four severity groups. To improve the visualization of the coupling focus at very low frequencies, an amplification between 0 and 0.05 Hz is depicted in the upper right corner for each figure. (**a**) No-OSA; (**b**) mild OSA; (**c**) moderate OSA; (**d**) severe OSA.

**Figure A1:**
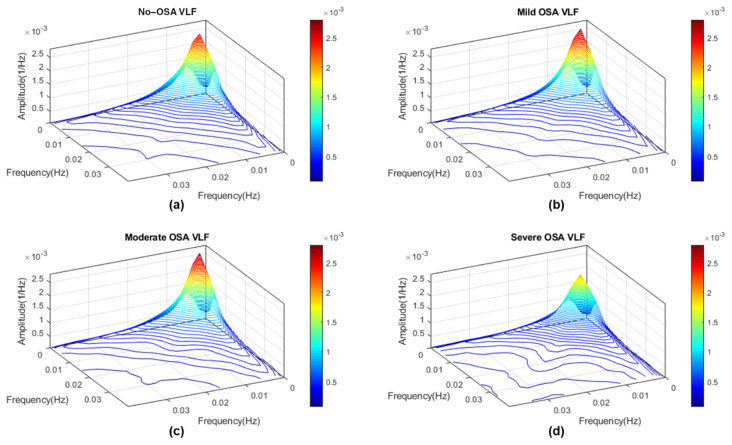
Averaged bispectrum magnitude in the very low frequency region (0–0.04 Hz) for the four severity groups in the training set. (**a**) No-OSA; (**b**) mild OSA; (**c**) moderate OSA; (**d**) severe OSA.

**Figure A2:**
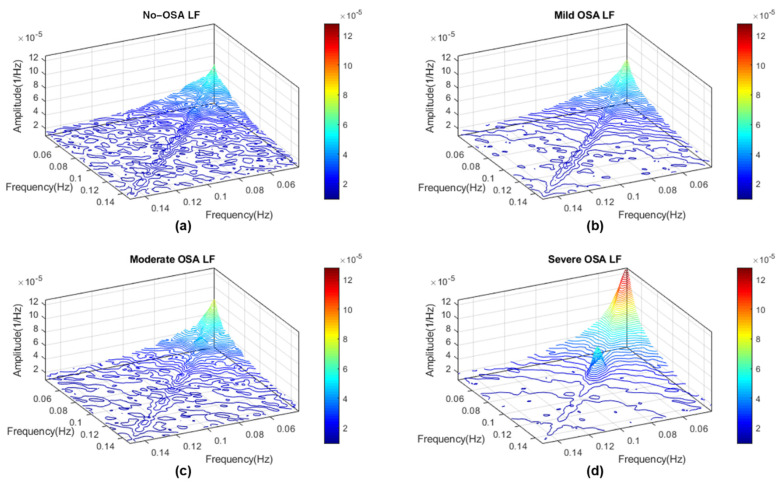
Averaged bispectrum magnitude in the low-frequency region (0.04–0.15 Hz) for the four severity groups in the training set. (**a**) No-OSA; (**b**) mild OSA; (**c**) moderate OSA; (**d**) severe OSA.

**Figure A3:**
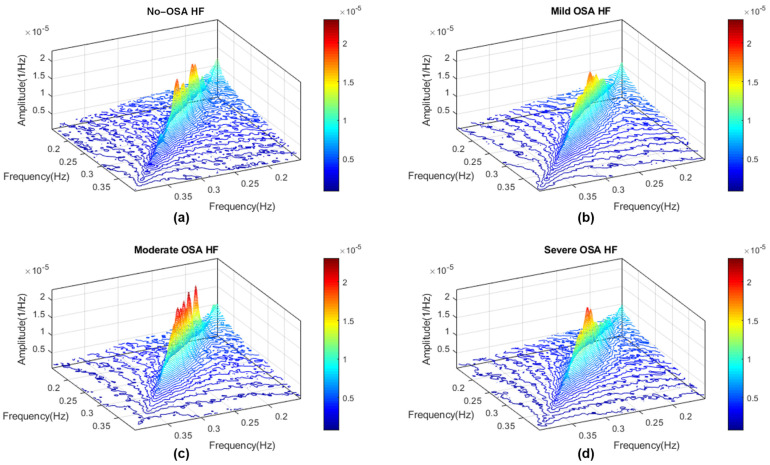
Averaged bispectrum magnitude in the high-frequency region (0.15–0.40 Hz) for the four severity groups in the training set. (**a**) No-OSA; (**b**) mild OSA; (**c**) moderate OSA; (**d**) severe OSA.

**Figure A4:**
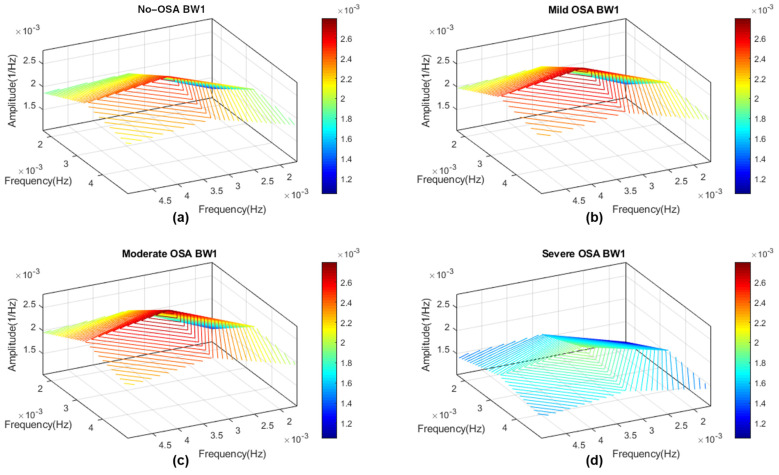
Averaged bispectrum magnitude in the BW1 frequency region (0.001–0.005 Hz) for the four severity groups in the training set. (**a**) No-OSA; (**b**) mild OSA; (**c**) moderate OSA; (**d**) severe OSA.

**Figure A5:**
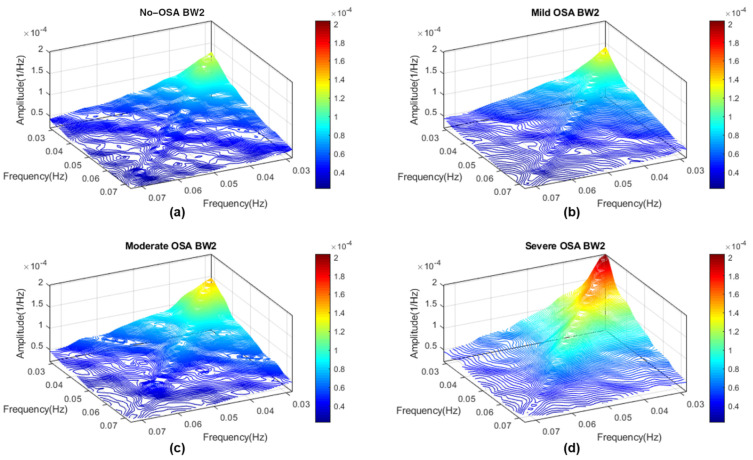
Averaged bispectrum magnitude in the BW2 frequency region (0.028–0.074 Hz) for the four severity groups in the training set. (**a**) No-OSA; (**b**) mild OSA; (**c**) moderate OSA; (**d**) severe OSA.

**Figure A6:**
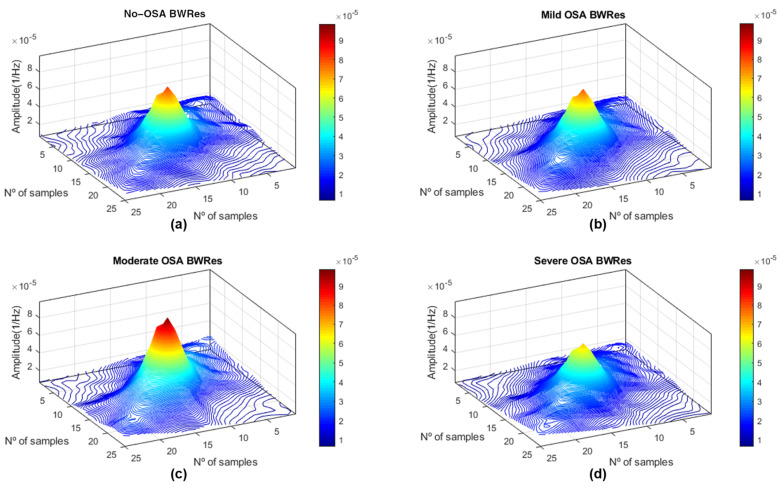
Averaged bispectrum magnitude in the BWRes frequency region (0.04 Hz around the respiratory peak inside HF) for the four severity groups in the training set. (**a**) No-OSA; (**b**) mild OSA; (**c**) moderate OSA; (**d**) severe OSA.

Therefore, in this study, six sub-band regions were assessed, three based on the classic HRV frequency ranges (VLF, LF and HF bispectral regions), and three based on the HRV OSA-related frequency ranges (BW1, BW2 and BWRes bispectral regions). To provide an overview, Figure 1 shows the averaged bispectrum magnitude in the training set for the four disease severity groups considered in the range 0–0.4 Hz. It can be observed that the no-OSA bispectral power is mainly concentrated below 0.02 Hz, and it spreads over a higher range of frequencies as the severity increases. The 3D bispectral region representations, averaged for each severity group, are shown in Appendix A Figure A1, Figure A2, Figure A3, Figure A4, Figure A5 and Figure A6.

### 3.3. Feature Extraction Stage

In order to characterize the bispectral regions under study, we computed features based on four different approaches: bispectral region amplitude, bispectral region entropy, bispectral region moment and weighted center of bispectrum (WCOB) region features. Furthermore, we introduced a new bispectral feature in this study.

As explained in Appendix A, BWRes is an adaptive frequency band based on the maximum value inside the HF range. It means that the frequency range is different for each subject, as it depends on the location of this peak. Therefore, contrary to the rest of the regions, BWRes might not be centered in the main diagonal of the bispectral matrix. Before feature extraction, we confirmed that this occurred in most of the subjects considered. As some of the features included in this study were computed over the diagonal of each region, the physiological meaning of the diagonal elements was lost (*f*_1_ ≠ *f*_2_); therefore, these features were not computed over the BWRes region in this study.

#### 3.3.1. Bispectral Region Amplitude Features

- Maximum amplitude (*B_max_*), measured as the maximum magnitude value inside each of the regions considered [46]:
  (3) $$B_{max}=\max\left(\left|B_{N}\left(f_{1},\;f_{2}\right)\right|\right),\;\;\;\;\;\;f_{1},\;f_{2}\;\in \;Ω,$$
  where Ω represents one of the six regions considered.
- Minimum amplitude (*B_min_*), measured as the minimum magnitude value inside each of the regions considered [46]:
  (4) $$B_{min}=\min\left(\left|B_{N}\left(f_{1},\;f_{2}\right)\right|\right),\;\;\;\;\;\;f_{1},\;f_{2}\;\in \;Ω$$
- Total bispectral power (*B_total_*), measured as the sum of all magnitudes inside each of the regions considered [46]:
  (5) $$B_{total}=\underset{f_{1},\;f_{2}\in \;Ω}{\sum }\left|B_{N}\left(f_{1},\;f_{2}\right)\right|.$$

This parameter allows measuring deviations from Gaussianity [46].

Following a similar tendency to the spectral approach [27], as severity increases, higher values of bispectral amplitude features are expected in regions related to OSA, such as BW2 or LF. Consequently, lower values with OSA in regions related to respiration, such as HF or BWRes, are also expected.

#### 3.3.2. Bispectral Entropy Features

- Normalized bispectral entropy (*BE_1_*), normalized squared bispectral entropy (*BE_2_*) and normalized cubed bispectral entropy (*BE_3_*). These parameters, based on Shannon’s entropy, quantify the irregularity of the bispectral distribution in each region and are computed as [29,34]
  (6) $$BE_{i}=-\underset{j\in \;Ω}{\sum }p_{j}\cdot \log\left(p_{j}\right),\;\;\;\;\;i=1,2,3$$
  where *p* is the magnitude distribution of the region elements:
  (7) $$p_{j}=\frac{{\left|B_{N}\left(f_{1},\;f_{2}\right)\right|}^{i}}{\sum_{f_{1},\;f_{2}\in \;Ω}{\left|B_{N}\left(f_{1},\;f_{2}\right)\right|}^{i}},\;\;\;\;\;\;i=1,2,3$$

The values of the bispectral entropies increase with the randomness of a process, meaning changes in the HRV irregularity as a result of OSA [30] would be captured by the bispectral entropies of the regions.

- Phase entropy (*PE*), which quantifies the phase regularity of the region [29]. *PE*, as with the bispectral entropies, is higher as the randomness of a process increases, meaning it would be zero for a harmonic, periodic and predictable process [34]. *PE* computation is performed applying Shannon’s entropy to the normalized distribution of the region phase angles [29,46]:
  (8) $$PE=-\underset{n\in \;Ω}{\sum }p\left(\Psi_{n}\right)\cdot \log\left(p\left(\Psi_{n}\right)\right)$$
  where
  (9) $$p\left(\Psi_{n}\right)=\frac{1}{L}\underset{f_{1},\;f_{2}\in \;Ω}{\sum }Ind\left(\varphi \left[B_{N}\left(f_{1},\;f_{2}\right)\right]\;\in \;\Psi_{n}\right),$$
  (10) $$\Psi_{n}=\left\{\varphi |-\pi +\frac{2\pi n}{N}\;\leq \varphi <-\pi +\frac{2\pi \;\left(n+1\right)}{N}\right\},\;\;\;\;\;n=0,1,\ldots ,N-1$$
  where Ind(·) is the indicator function (equal to 1 if *φ* is within the range of histogram bins $\Psi_{n}$), *φ* is the phase angle of the region, and *N* is the bin number of the histogram.

#### 3.3.3. Bispectral Region Moment Features

- The sum of the logarithmic magnitude values of the region (*H*_1_), sum of the logarithmic magnitude values of the diagonal of the region (*H*_2_) and first- and second-order spectral moments of the magnitude values of the diagonal elements of the region (*H*_3_ and *H*_4_, respectively). These features were included as they allow characterizing the nonlinearity of the regions and are computed as follows [46]:
  (11) $$H_{1}=\underset{f_{1},\;f_{2}\in \;Ω}{\sum }\log\left(\left|B_{N}\left(f_{1},\;f_{2}\right)\right|\right).$$
  (12) $$H_{2}=\underset{f_{k},\in \;\;\Gamma_{diag}}{\sum }\log\left(\left|B_{N}\left(f_{k},\;f_{k}\right)\right|\right).$$
  (13) $$H_{3}=\underset{f_{k},\in \;\;\Gamma_{diag}}{\sum }k\cdot \log\left(\left|B_{N}\left(f_{k},\;f_{k}\right)\right|\right).$$
  (14) $$H_{4}=\underset{f_{k},\in \;\;\Gamma_{diag}}{\sum }{\left(k-H3\right)}^{2}\cdot \log\left(\left|B_{N}\left(f_{k},\;f_{k}\right)\right|\right)$$

Those children suffering from OSA would be expected to present an increased bispectral power concentration in the region defined by frequency ranges related to the occurrence of apneic events (BW2). This would mean an increase in the phase coupling between the frequency components of this region and, accordingly, higher nonlinearity [30,46]. Therefore, OSA children would be expected to present higher values of bispectral region moment features in regions related to OSA, and lower values in the respiratory-related regions.

#### 3.3.4. Bispectral WCOB Features

- WCOB allows reflecting the interaction of different frequency components through the assignment of a weight to each bispectral point of the region [46]. The weighted center of each region is composed of two vectors, *f1m* and *f2m*, which indicate the coupling focus of the region as a summary of the frequency interaction [46]. Those components of WCOB are computed as [46]
  (15) $$f1m=\frac{\sum_{f_{1},\;f_{2}\in \;Ω}f_{1}\cdot B_{N}\left(f_{1},\;f_{2}\right)}{\sum_{f_{1},\;f_{2}\in \;Ω}B_{N}\left(f_{1},\;f_{2}\right)},$$
  (16) $$f2m=\frac{\sum_{f_{1},\;f_{2}\in \;Ω}f_{2}\cdot B_{N}\left(f_{1},\;f_{2}\right)}{\sum_{f_{1},\;f_{2}\in \;Ω}B_{N}\left(f_{1},\;f_{2}\right)}$$

WCOB parameters are associated with bispectral peak values, with decreases in *f1m* and *f2m* values implying an activity shift towards lower frequencies [46]. Hence, as OSA children are expected to present higher activity in bispectral regions related to apneic events, their WCOB would be centered around these regions.

#### 3.3.5. Relative Power of the Diagonal, a Novel Bispectral Feature

- The relative power of the diagonal (*RP_Diag_*), computed as the sum of the bispectral amplitudes of the diagonal elements of the region, after a normalization applied over the whole diagonal. This novel parameter evaluates the relative bispectral magnitude value inside the diagonal of the region with respect to the complete bispectral diagonal magnitude:
  (17) $$RP_{Diag}=\underset{f_{k}\;\in \;\Gamma_{diag}}{\sum }\left|Diag_{N}\left(f_{k}\right)\right|,$$
  where $\Gamma_{diag}$ represents the diagonal elements of one of the regions considered except BWRes, and $Diag_{N}$ is the normalized bispectral diagonal after the normalization performed such that
  (18) $$Diag_{N}\left(f_{k}\right)=\frac{Diag\left(f_{k}\right)}{DP},\;\;\;\;\;f_{k}=0,\cdots ,\;f_{N}$$
  where *Diag* is the diagonal of the bispectral matrix, and *DP* is the diagonal power, measured as the sum of all amplitudes of the *Diag* elements.

The diagonal elements of a region are a particular case of the bispectral matrix when *f_1_ = f_2_*; therefore, this parameter, as well as *H*2, is intended to measure the phase coupling between the harmonic components of HRV signals, such that *f*_3_ = 2*f*_1_ and *φ*_3_ = 2*φ*_1_ [30,46].

*RP_Diag_* and *H*2 present two important differences. First, a normalization over the whole diagonal is applied in this novel feature. As a result of this normalization, the sum of all bispectral amplitudes of the diagonal elements is equal to 1, meaning *RP_Diag_* evaluates the proportion of the total diagonal bispectral power contained in the region. Then, as the normalization is scaling the values of the diagonal elements, we use a linear scale to compute the sum of the relative power instead of the logarithmic scale applied in *H*2. The rationale of this parameter lies in the normalization considering all of the frequency range. When OSA occurs, there is an alteration in the synchronization of the heart rhythm [30], leading to a redistribution of HRV activity to frequency components associated with the occurrence of apneic events. Thus, the normalization applied here considers not only the bispectral power in the diagonal of the region evaluated but also that in other diagonal elements. This influence of the redistribution to other frequency ranges is lost when applying a logarithmic scale.

Table 2 summarizes the bispectral features computed in every region under study.

### 3.4. Feature Selection Stage

Once each region was characterized with the features detailed in Table 2, we constructed two different optimal feature subsets (classic and OSA-specific bands) via the FCBF algorithm. This method, which has been previously demonstrated to be useful in pediatric OSA diagnosis [42,45,46,51], allows creating a non-redundant and relevant feature set based on the symmetrical uncertainty [50].

We performed the selection stage over 1000 bootstrap replicates from the training dataset in order to obtain generalizable and non-dependent subsets [52]. Those features selected more than 500 times were chosen to form the optimal subsets [42,45,51].

### 3.5. Classification Stage

Similar to previous pediatric OSA diagnosis studies, we conducted the classification stage using MLP neural networks [42,46,51]. These neural networks are typically formed by an input layer, a hidden layer and an output layer, each one composed of a different number of neurons, called perceptrons [53]. Each perceptron of an MLP network layer is connected to all the perceptrons from the next layer, with a weight associated with this connection [53]. The number of perceptrons in the first layer is equal to the number of input features. The number of perceptrons in the output layer depends on the objective of the network. We performed binary classification for the three severity thresholds (1, 5 and 10 e/h), as in our previous work [27]. This implies three different MLP neural networks for each feature subset, with one perceptron in the output layer providing the posterior probability of belonging to the severity group considered at each case [53]. The number of perceptrons in the hidden layer (*N_H_*) is a parameter to be optimized. To deal with overfitting, we also introduced a regularization parameter (*λ*) in the tuning of the network weights, which were randomly initialized [53].

The optimization of the hidden layer design parameters (*N_H_* and *λ*) was performed, again, by means of 1000 bootstrap replicates from the training dataset, but different from the replicates employed in the feature selection stage. We computed Cohen’s kappa (*k*) for each *N_H/_λ* combination and selected those values where *k* was maximum [42,46,51].

Thus, six MLP neural networks were optimized, one for each feature subset and severity threshold.

### 3.6. Statistical Analysis

In the training set, the features included in this study did not pass normality and homoscedasticity tests. For this reason, we assessed statistically significant differences (*p*-value < 0.01 after applying Bonferroni correction) between bispectral features from OSA severity groups through the non-parametric Kruskal–Wallis test. To provide a visual representation of these differences, along with the distribution followed by the features in each severity group, boxplots for each selected feature were also constructed.

Subsequently, we conducted a correlation analysis. To this effect, relationships between the features selected and some polysomnographic indices were evaluated using Spearman’s partial correlation coefficient (*ρ_S_*), controlling the possible effect of age. The polysomnographic indices, related to OSA, as well as sleep structure and quality, were the same as those in [27]: AHI, Obstructive AHI (OAHI), obstructive apnea index (OAI), oxygen desaturation index (ODI), wake after sleep onset (WASO), number of awakenings during total sleep time (#Awakenings), percentage of total sleep spent in sleep stages N1, N2, N3 and rapid eye movement (%N1, %N2, %N3 and %REM, respectively) and total arousal index per hour of sleep (TAI). Correlation analysis was performed on the test set.

Finally, after the optimization of the MLP neural networks in the training set, the diagnostic performances of each individual selected feature and optimized model were evaluated in the test set in terms of sensitivity (Se), specificity (Sp), accuracy (Acc) and area under the receiver operating characteristic curve (AUC).

## 4. Results

### 4.1. Feature Selection in the Training Set

**Figure 2:**
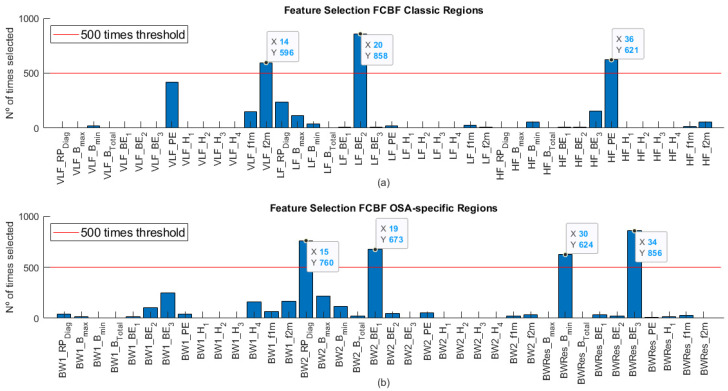
Features selected in each feature set after applying FCBF algorithm over 1000 bootstrap replicates of the training set. (**a**) Features selected in the classic region set; (**b**) features selected in the OSA-specific region set.

We conducted two feature selection processes. As Table 2 shows, the number of input features for the classic bispectral region set was 42, while in the case of the OSA-specific region set, there were up to 38 features. The feature selection through FCBF allowed reducing the amount of redundant information while assessing feature complementarity. Figure 2 shows the number of times that each feature was selected by the algorithm in the 1000 bootstrap replicates for both cases. In the case of the classic regions (Figure 2a), it can be observed that the optimum subset (BISP_Classic_) was formed by three features, one of each region: *VLF_f2m*, *LF_BE_2_* and *HF_PE*. Regarding the OSA-specific region set (Figure 2b), the optimum subset (BISP_Specific_) was composed of four features selected more than 500 times: *BW2_RP_Diag_*, *BW2_BE_1_*, *BWRes_B_min_* and *BWRes_BE_3_*. None of the BW1 region features considered were selected over 500 times.

### 4.2. Descriptive Analysis of the Features Selected

**Figure 3:**
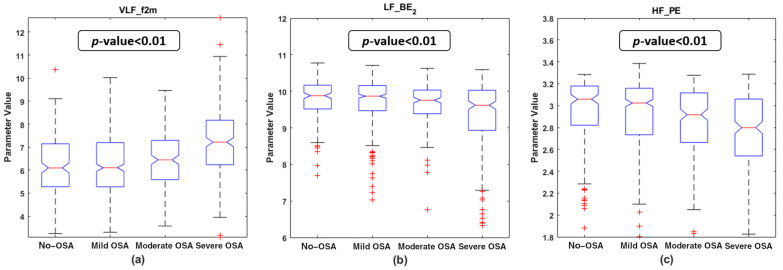
Boxplot distribution of the features selected in the bispectral classic region feature subset for the four OSA severity groups in the training set. The *p*-value obtained with the Kruskal–Wallis test is shown in each subplot. (**a**) *VLF_f2m* boxplots and *p*-value; (**b**) *LF_BE_2_* boxplots and *p*-value; (**c**) *HF_PE* boxplots and *p*-value.

**Figure 4:**
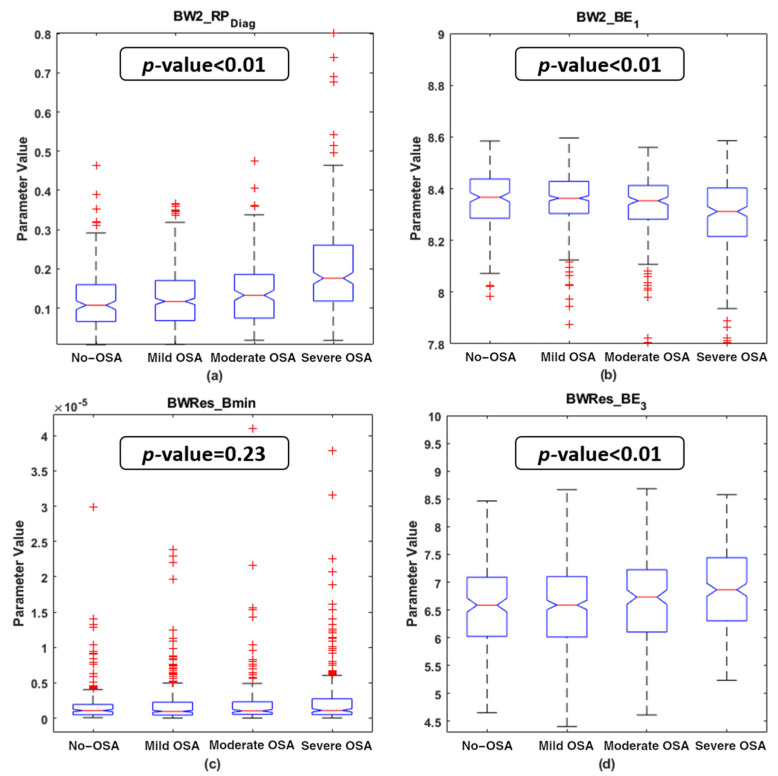
Boxplot distribution of the features selected in the bispectral OSA-specific region feature subset for the four OSA severity groups in the training set. The *p*-value obtained with the Kruskal–Wallis test is shown in each subplot. (**a**) *BW2_RP_Diag_* boxplots and *p*-value; (**b**) *BW2_BE_1_* boxplots and *p*-value; (**c**) *BWRes_B_min_* boxplots and *p*-value; (**d**) *BWRes_BE_3_* boxplots and *p*-value.

Figure 3 and Figure 4 show the boxplot distributions of the four OSA severity groups for the features selected in both the BISPc_lassic_ and BISP_Specific_ subsets, respectively. The *p*-value resulting from the Kruskal–Wallis test is also depicted in these figures. It can be appreciated that, in the BISP_Classic_ subset, *VLF_f2m* experienced an increase with OSA severity, while a decrease in *LF_BE_2_* and *HF_PE* occurred as the OSA severity increases. For the features included in BISP_Specific_, there was a clear rise in the *BW2_RP_Diag_* values, along with a slight increase in *BWRes_BE_3_* with OSA severity. In contrast, the *BW2_BE_1_* values experienced a decrease with the disease. *BWRes_B_min_* was the only parameter showing an unclear tendency among the severity groups, which led it to be the only one that did not show statistically significant differences. The remaining six parameters showed statistically significant differences among the four OSA severity groups (*p*-value < 0.01 after Bonferroni correction).

### 4.3. MLP Network Optimization and Training

After extraction of the optimum feature subsets, six MLP models were optimized: three models with BISPc_lassic_ features as input (MLP1_Classic_, MLP5_Classic_ and MLP10_Classic_, with AHI = 1, 5 and 10 e/h as thresholds for binary classification, respectively), and three models with BISP_Specific_ as input features (MLP1_Specific_, MLP5_Specific_ and MLP10_Specific_, with AHI = 1, 5 and 10 e/h as a threshold for binary classification, respectively). For each model, *N_H_* varied from 2 to 20 in steps of 1, and from 22 to 50 in steps of 2. Similarly, λ varied from 0.5 to 10 in steps of 0.5. Each *N_H/_λ* pair resulted in an averaged *k* through 1000 bootstrap replicates of the training set; therefore, we selected the *N_H_/λ* combination with the higher averaged *k*. *N_H_* = 2 and *λ* = 5 were the optimized design parameters selected in four out of six MLP models: MLP1_Classic_, MLP5_Classic_, MLP5_Specific_ and MLP10_Specific_. For the MLP10_Classic_ model, the optimized design parameters were *N_H_* = 34 and *λ* = 5. Finally, the optimized parameters in MLP1_Specific_ were *N_H_* = 38 and *λ* = 5.

### 4.4. Correlation Analysis in the Test Set

The results of the correlation study are shown in Table 3. Although the values of |*ρ_S_*| obtained were generally low, some of the correlations evaluated were statistically significant (*p*-value < 0.01 after Bonferroni correction). *VLF_f2m* and *BW2_RP_Diag_* showed similar behaviors, with a statistically significant positive *ρ_S_* with the four respiratory indices (AHI, OAHI, OAI and ODI) and TAI, as well as a negative *ρ_S_* with %REM. In the opposite way, *BW2_BE_1_* obtained a negative *ρ_S_* with the four respiratory indices and TAI. *LF_BE_2_* also reached a statistically significant negative *ρ_S_* with AHI, OAHI and TAI. *BW2_RP_Diag_* reached the highest absolute correlation values among almost all of these statistically significant correlations, only being equaled by the *ρ_S_* reached between *VLF_f2m* and OAHI. None of the selected BWRes features nor *HF_PE* obtained statistically significant correlations with any of the polysomnographic indices considered in this study.

### 4.5. Diagnostic Ability Assessments

Table 4 shows the diagnostic performance obtained in the test set by each feature individually, along with the classification results reached by each MLP model optimized for the three severity thresholds.

Regarding the individual performance, in the 1 e/h threshold, *BW2_RP_Diag_* obtained the highest results in terms of Acc and AUC. For the 5 e/h threshold, again, *BW2_RP_Diag_* showed a higher Acc than the other features selected, being only slightly surpassed by *VLF_f2m* in terms of AUC. When considering 10 e/h as severity cutoff for binary classification, *LF_BE_2_* was the feature showing the higher Acc, but with a lower AUC and a more unbalanced Se/Sp pair than *BW2_RP_Diag_*.

All the individual diagnostic yields were outperformed by the MLP models. The MLP models formed by the OSA-specific region features obtained the highest results of this study in terms of Acc and AUC in the 1 and 5 e/h thresholds. The MLP10_Classic_ model was the only one that achieved a higher diagnostic performance than the OSA-specific models in terms of Acc and AUC at the cost of a strongly unbalanced Se/Sp pair and a very low Se value (43.5%).

## 5. Discussion

In this work, bispectral analysis of HRV signals, a process performed for the first time in pediatric OSA research, was conducted herein. Features from bispectral HRV regions based on the classic and OSA-specific frequency ranges were extracted to assess their usefulness in the characterization and diagnosis of pediatric OSA. In both types of regions separately, the selected features showed their complementarity, and the models constructed achieved a high diagnostic performance. The OSA-specific region models generally outperformed the classic ones, highlighting the importance of these novel bispectral features in the study of pediatric OSA.

### 5.1. Physiological Interpretation of the Features Selected

The averaged normalized bispectrum in the training set shown in Figure 2 serves as a summary of the degree of phase coupling in the frequency range 0–0.4 Hz, covering all the regions considered. The bispectral power is mainly concentrated under 0.02 Hz in the no-OSA subjects, and then it spreads with severity to higher frequencies. This coupling focus is markedly lower in the severe OSA group, as it can be observed in the amplification depicted in each upper right corner of the representations from Figure 2. It reflects an increase in the linearity and Gaussianity of HRV signals at very low frequencies due to apneic events [29]. This shift to higher frequencies is also reflected in the selected feature *VLF_f2m*. The values of *VLF_f2m* increase with OSA severity, as depicted in Figure 3a. This means that the focus of coupling in the VLF range is displaced by apneic events, generating higher HRV activity at higher frequencies [54]. This feature makes sense, as the higher frequencies of the VLF range (0–0.04 Hz) overlap with the lower frequencies of BW2 (0.028–0.074 Hz), which has been defined as the frequency range related to the duration of apneic events [27].

Inside this BW2 apneic-related region, *BW2_RP_Diag_* was one of the two features selected. This parameter, as with *VLF_f2m*, showed an increasing tendency with OSA severity (Figure 4a). As explained in the Methods section, *RP_Diag_* is intended to measure phase coupling between the harmonic components (*f*_3_ = 2*f*_1_ and *φ*_3_ = 2*φ*_1_) of the HRV. The increment in *BW2_RP_Diag_* with OSA severity would indicate increasing nonlinear interactions between those harmonics of OSA-affected children. Consequently, there is an increment in less random/more periodic harmonics in HRV signals inside the BW2 region due to apneic events. The increase in *VLF_f2m* and *BW2_RP_Diag_* with OSA severity is supported by the correlation study, showing statistically significant correlations with all respiratory indices, as well as with TAI. Furthermore, *BW2_RP_Diag_* generally obtained the highest individual diagnostic performance. Taken together, these facts highlight the importance of analyzing the BW2 bispectral region in the field of pediatric OSA, especially characterized through our new proposed parameter *RP_Diag_*.

The entropy features also showed their usefulness to characterize the bispectral regions of the HRV. Three entropies of the bispectral amplitude distribution were selected: *BE_1_* inside the BW2 region, *BE_2_* inside the LF region and *BE_3_* inside BWRes. These parameters measure the irregularity of the HRV from the bispectral distribution in each region, with the inclusion of quadratic and cubic components scaling the differences in the bispectral amplitude [29]. Bispectral distributions averaged for each severity group in these three regions are shown in Figure A2 (LF region), A4 (BW2 region) and A5 (BWRes region). In the case of *BW2_BE1*, a decrease with severity was observed, reflecting a reduction in irregularity with apneic events in the HRV components linked to the frequencies of this region. As a result of apneic events, it can be appreciated that the bispectral amplitude in BW2 is more concentrated at low frequencies in the severe group (Figure A4d) and starts to distribute more randomly to other frequencies as OSA decreases. This may be due to the aforementioned increment in the less random harmonics in this range due to OSA, leading to the reduction with severity experimented in *BW2_BE_1_* (Figure 4b). In the case of *LF_BE_2_*, this parameter also decreases with the severity of the disease, again reflecting a reduction in irregularity with OSA in the HRV associated with this frequency region. Figure A2 points to the fact that the bispectral power distribution of no-OSA subjects is more dispersed over the whole LF region and starts to concentrate at lower frequencies as OSA severity increases. Interestingly, this parameter only showed negative statistically significant correlation values when considering respiratory indices that include hypopneas (AHI and OAHI). It seems that, as apneas are less frequent than hypopneas in the database under study, the only effect of apneas is not enough to decrease the bispectral HRV irregularity associated with the LF frequency range. Similarly, collective apneic effects are better captured with the inclusion of the quadratic amplitude, suggesting that *BE_2_* is more accurate in detecting alterations in the bispectral distribution of LF as a result of all apneic events. Regarding *BWRes_BE_3_*, Figure 4d shows that no-OSA, mild OSA and moderate OSA subjects presented lower values than the severe OSA group. As it can be seen in Figure A6, there is a higher bispectral power concentration around the respiratory peak for the first three severity groups, and a lower concentration for severe OSA, whose distribution spreads over other frequencies. The lower coupling around the respiratory peak in the severe group makes sense as OSA results in a redistribution in the bispectral power to frequency ranges related to apneic events, such as the BW2 region. These are milder differences than those observed in BW2 and LF; therefore, an increase in HRV irregularity due to OSA appears to be better captured through *BE_3_* from BWRes.

In addition to the bispectral entropies, *PE* was also selected in the HF range. This parameter showed a decreasing tendency with OSA severity (Figure 3c). As a reduction in *PE* indicates that a process becomes less random [34], this result suggests that OSA alterations lead to a reduction in the irregular behavior of the HRV phase along the HF region. The fact that every entropy measure included in this study was selected, at least, in one region highlights the importance of the entropy features when analyzing the bispectral HRV distribution in pediatric OSA.

*BWRes_B_min_* was the remaining feature selected. The normalization applied over each bispectral matrix allowed *B_min_* to estimate the minimum coupling within this region [46]. Despite the unclear tendency and the absence of differences obtained in this parameter (Figure 4c), *BWRes_B_min_* was selected by the algorithm. This implies that *BWRes_B_min_* contains information that is complementary to the other features selected in the OSA-specific region feature subset.

Although BW1 showed its potential utility in the spectral analysis [27], none of the features included in this region were selected by the FCBF algorithm a number of times above the threshold established. This suggests that, when analyzing pediatric OSA alterations in the HRV bispectrum, the assessment of the BW2 and BWRes regions would be enough to characterize OSA effects. Similarly, the features related to bispectral moments were not selected either, probably due to the redundancy when introducing *RP_Diag_*, which seems to be more accurate in the characterization of apneic alterations.

### 5.2. Diagnostic Performance of the Bispectral Models

In this study, the information extracted from the bispectral HRV regions obtained an overall high diagnostic performance both in the classic and OSA-specific bispectral region models in the test set. The results obtained in the MLP models outperform the individual diagnostic yield, highlighting the utility of the FCBF algorithm and MLP neural networks to assess complementarity between features and to diagnose pediatric OSA, respectively. When comparing classic against OSA-specific region models, the latter generally obtained a higher performance. In the 1 e/h severity cutoff, despite the unbalanced Se/Sp pair, MLP1_Specific_ obtained a higher Acc (63.4% versus 54.7% from the MLP1_Classic_ model) and a higher AUC (0.627 versus 0.600). In the 5e/h threshold, the Acc obtained by MLP5_Specific_ and MLP5_Classic_ was 81.0% in both cases, but with the specific model, showing a more balanced Se/Sp, a higher AUC was found (0.774 versus 0.791). Finally, in the 10e/h severity cutoff, MLP10_Classic_ obtained the highest Acc and AUC from the study. However, the Se/Sp pair was strongly unbalanced, with a very low Se value of 43.5%. Nevertheless, the MLP10_Specific_ model, at the cost of a very slight reduction in terms of Acc and AUC (89.3% versus 91.7%, and 0.847 versus 0.841, respectively), resulted in a more balanced Se/Sp pair (66.7%/91.6%). These results reinforce the conclusion from our previous work [27] about the importance of analyzing OSA-specific HRV frequency ranges whenever pediatric OSA is under study.

### 5.3. Comparison with Previous Work

As far as we know, this is the first study where HRV bispectral analysis of pediatric OSA was conducted. In this sense, a direct comparison of classification results with other research studies using an HRV bispectral approach is not possible. However, HRV analysis to diagnose pediatric OSA has been previously performed. A research group carried out three classification studies [26,55,56], where they derived HRV features from decreases in the amplitude fluctuations in the photoplethysmography signal. In their studies, they included a population of 21 children (10 children with OSA and 11 controls) and obtained an Acc ranging from 73.3% to 80%, Se from 62.5% to 87.5% and Sp from 71.45% to 85.7%, when considering HRV features only. The highest results obtained in the present work outperform these diagnostic results in terms of Acc and Sp, with Se in the same range. Nonetheless, the difference in the child population, along with the different criteria followed to establish OSA, makes it difficult to perform a more comprehensive comparison. The study developed by Cohen and de Chazal [57] was the first study conducting an automated classification of children with OSA using only the HRV signal. Notwithstanding, this classification was based on detecting apneic events, rather than global classification of each subject, meaning a comparison with the diagnostic results of the present study is not possible [57].

Thus, our previous study [27] is the only one that establishes a benchmark with which we can compare the diagnostic performance reached in the present study. Furthermore, the comparison of these results is direct as the database, the distribution of training/test sets and the criterion of OSA diagnosis were the same. In the previous work, we computed the individual diagnostic performances of each relative power (RP) from the power spectral density in the VLF, LF, HF, BW1, BW2 and BWRes bands, and the LF/HF ratio too. Then, we constructed two linear discriminant analysis (LDA) models to assess the joint diagnostic yield of the relative powers in the classic bands versus the OSA-specific bands. Following this methodology, the best classification outcomes obtained in terms of Acc and AUC using 1 e/h as the threshold for binary classification were 59.2% Acc (RP_BW1_ individually) and 0.592 AUC (LDA band of interest model). With 5 e/h as the severity threshold, the highest results were 76.6% Acc (RP_BW2_) and 0.688 AUC (LDA band of interest model). Finally, in the 10 e/h severity cutoff, the highest results reached were 82.8% Acc and 0.796 AUC (both using the LDA band of interest model). It can be observed from Table 4 that, in the present work, the best results achieved with the MLP optimized models surpassed, by far, those achieved previously, and even some of the individual bispectral features eventually outperformed several of these results. It can be argued that the improvement in the diagnostic outcomes may be mediated by the increased complexity of the classification algorithm. To deal with this issue, and also for a fair comparison, we have included in Appendix B the results obtained using the same classification methodology as in [27]. Table A1 shows the classification results obtained including the features selected from each approach using an LDA classifier. The results obtained with both the LDA_Classic_ and LDA_Specific_ models also outperform the diagnostic performance obtained in the previous work, being only surpassed in terms of Acc in the 1 e/h threshold. Thus, the diagnostic utility of the features extracted from the bispectral HRV region analysis is clearly demonstrated, with the MLP models reaching the highest diagnostic performance in the literature when using HRV features exclusively to generate an automated classification of pediatric subjects into the presence or absence of OSA and to estimate the severity grouping. Moreover, the higher diagnostic performance reached by the bispectral analysis highlights the usefulness of this analysis in the pediatric OSA context, which seems to be more accurate than traditional frequency analysis to evaluate HRV alterations. The presence of HRV nonlinear dynamics demonstrated in Appendix C and captured through the HRV bispectrum estimation may be behind this improvement over the traditional techniques.

Finally, although the results of the diagnostic performance from studies using physiological data from different sources must be carefully compared, it is interesting to contrast our results with the meta-analysis of a recently published systematic review [14]. In this work, Gutiérrez-Tobal et al. gathered the pooled Se and Sp results from nineteen studies of machine learning methods to diagnose pediatric OSA that fulfilled their eligibility criteria [14]. A meta-analysis was performed for the same OSA severity thresholds that were employed here, which obtained an Se of 84.9%, 71.4% and 65.2% for the 1, 5 and 10 e/h cutoffs, respectively. Similarly, the meta-analysis obtained an Sp of 49.9%, 83.2% and 93.1% for the 1, 5 and 10 e/h cutoffs, respectively. Table 4 shows that the MLP models presented here for both classic and specific approaches obtained a diagnostic performance in the same range for Sp in the 5 and 10 e/h cutoffs, and also MLP10_Specific_ obtained similar Se results. It is worth noting that, among the studies included in the meta-analysis, none of them considered HRV signals. Furthermore, the sample size from seventeen out of nineteen studies included in the systematic review was markedly smaller than the databases analyzed here, only being surpassed by the cohort included in the studies of Hornero et al. [42] and Vaquerizo-Villar et al. [58]. Therefore, these considerations along with the similar diagnostic performance reached in the 5 and 10 e/h severity thresholds reinforce the support for the use of HRV bispectral analysis as a potential alternative to overnight PSG for pediatric OSA diagnosis.

### 5.4. Limitations and Outlook

Despite the high diagnostic performance obtained with the methodology followed in this study, some limitations deserve mention. First, although the sample size included is markedly large, accounting for 1738 overnight HRV recordings, some imbalance between severity groups is apparent. In this sense, an increase in the population included in future studies, trying to balance the severity groups’ distribution, would be desirable to raise the robustness and generalizability of our results.

Additionally, none of the features extracted from the region bound by BW1 frequencies (0.001–0.005 Hz) were selected. This frequency range has been linked to sleep fragmentation due to OSA, and its usefulness in spectral analysis has been demonstrated [27]. Thus, a combination of features from this region with features derived from different approaches would be performed in future studies to assess if BW1 OSA alterations are really reflected in bispectral analysis, and also if they contain non-redundant information on the features presented here.

A previous study performing bispectral HRV analysis in the adult OSA context only analyzed the non-redundant bispectral region [30]. In this sense, despite the well-known differences between children and adults in the OSA context, the high diagnostic performance achieved here in analyzing classic and OSA-specific bispectral regions serves as a motivation to search for adult OSA-specific frequency ranges. Consequently, a replication of the methodology carried out here to extract OSA-specific regions in adults and its analysis in the frequency and bispectral domains is one of our future research aims.

Additionally, the highly satisfactory results achieved illustrate the utility of bispectral HRV analysis to characterize and diagnose pediatric OSA, outperforming the previous spectral analysis diagnosis yield [27]. Therefore, the binary classification performed serves as a first step, and further explorations of more complex predictive models, as well as estimation of the AHI (regression), instead of binary classification, are also some of our future research aims.

Finally, despite the high diagnostic performance achieved here through bispectral analysis and the MLP models constructed, the promising results obtained by deep learning techniques in healthcare issues in recent years highlight the potential utility of these methods to automate the diagnosis of pediatric OSA [14]. Accordingly, in trying to increase the diagnostic performance, the inclusion of deep learning methods in the pediatric OSA context is a future research need.

## 6. Conclusions

To the best of our awareness, this is the first work where bispectral HRV analysis has been conducted to characterize and diagnose pediatric OSA. Our methodology allowed us to obtain two feature subsets, one containing information regarding bispectral regions based on classic HRV frequency ranges, and the other one with OSA-specific bispectral regions. Those subsets were formed by features containing complementary information about alterations in the non-Gaussianity, nonlinearity and irregularity behavior of the HRV due to OSA. Among the features selected, a novel bispectral measure presented here, *RP_Diag*, showed its utility, generally achieving the highest individual diagnostic performance as well as the highest correlations with polysomnographic indices. Furthermore, the MLP models outperformed the previous results of diagnostic performance based on spectral analysis, with the MLP1_Specific_, MLP5_Specific_ and MLP10_Classic_ models achieving the highest diagnostic yield from the study for each severity cutoff. These results highlight the usefulness of bispectral HRV analysis in the pediatric OSA context, especially when analyzing bispectral regions bounded by OSA-specific frequency ranges. Thus, we conclude that information extracted from HRV bispectra allows for the characterization and diagnosis of pediatric OSA, leading us to propose this approach as a potential alternative to PSG.

## Figures and Tables

**Table 1:** Clinical and demographic data from children included in this study.

	All	Training Set (UofC)	Test Set (CHAT)
Subjects (*n*)	1738	981	757
Age (years)	6.4 [3.3]	6.0 [6.0]	7.0 [2.4]
Males (*n*)	962 (55.35%)	602 (61.37%)	360 (47.95%)
BMI (kg/m^2^)	17.63 [5.37]	18.02 [5.86]	17.28 [4.64]
AHI (e/h)	2.23 [5.27]	3.8 [7.76]	1.46 [2.07]
AHI ≥ 1 (e/h)	1309 (75.31%)	808 (82.36%)	501 (66.18%)
AHI ≥ 5 (e/h)	519 (29.86%)	407 (41.49%)	112 (14.80%)
AHI ≥ 10 (e/h)	298 (17.15%)	229 (23.34%)	69 (9.11%)

Data are shown as median [interquartile range] or *n* (percentage). BMI: body mass index; AHI: Apnea–Hypopnea Index; UofC: University of Chicago; CHAT: Childhood Adenotonsillectomy Trial.

**Table 2:** Summary of the bispectral features initially computed in each region. Features related to the diagonal of the region were excluded in the BWRes region.

	Classic Region Feature Set			OSA-Specific Region Feature Set		
Features	VLF	LF	HF	BW1	BW2	BWRes
*RP_Diag_*	*VLF_RP_Diag_*	*LF_RP_Diag_*	*HF_RP_Diag_*	*BW1_RP_Diag_*	*BW2_RP_Diag_*	*-*
*B_max_*	*VLF_B_max_*	*LF_B_max_*	*HF_B_max_*	*BW1_B_max_*	*BW2_B_max_*	*BWRes_B_max_*
*B_min_*	*VLF_B_min_*	*LF_B_min_*	*HF_B_min_*	*BW1_B_min_*	*BW2_B_min_*	*BWRes_B_min_*
*B_Total_*	*VLF_B_Total_*	*LF_B_Total_*	*HF_B_Total_*	*BW1_B_Total_*	*BW2_B_Total_*	*BWRes_B_Total_*
*BE_1_*	*VLF_BE_1_*	*LF_BE_1_*	*HF_BE_1_*	*BW1_BE_1_*	*BW2_BE_1_*	*BWRes_BE_1_*
*BE_2_*	*VLF_BE_2_*	*LF_BE_2_*	*HF_BE_2_*	*BW1_BE_2_*	*BW2_BE_2_*	*BWRes_BE_2_*
*BE_3_*	*VLF_BE_3_*	*LF_BE_3_*	*HF_BE_3_*	*BW1_BE_3_*	*BW2_BE_3_*	*BWRes_BE_3_*
*PE*	*VLF_PE*	*LF_PE*	*HF_PE*	*BW1_PE*	*BW2_PE*	*BWRes_PE*
*H_1_*	*VLF_H_1_*	*LF_H_1_*	*HF_H_1_*	*BW1_H_1_*	*BW2_H_1_*	*BWRes_H_1_*
*H_2_*	*VLF_H_2_*	*LF_H_2_*	*HF_H_2_*	*BW1_H_2_*	*BW2_H_2_*	*-*
*H_3_*	*VLF_H_3_*	*LF_H_3_*	*HF_H_3_*	*BW1_H_3_*	*BW2_H_3_*	*-*
*H_4_*	*VLF_H_4_*	*LF_H_4_*	*HF_H_4_*	*BW1_H_4_*	*BW2_H_4_*	*-*
*f1m*	*VLF_f1m*	*LF_f1m*	*HF_f1m*	*BW1_f1m*	*BW2_f1m*	*BWRes_f1m*
*f2m*	*VLF_f2m*	*LF_f2m*	*HF_f2m*	*BW1_f2m*	*BW2_f2m*	*BWRes_f2m*

**Table 3:** Results of the partial correlation study in the test set between features selected for each subset and the polysomnographic indices considered.

**BISP_Classic_ Features**								
**PSG Index**	**VLF_f2m**		***LF_BE_2_***		***HF_PE***			
	***ρ_S_***	***p*-Value**	***ρ_S_***	***p*-Value**	***ρ_S_***	***p*-Value**		
AHI	**0.274**	**<<0.01**	**−0.185**	**<<0.01**	−0.112	0.002 *		
OAHI	**0.261**	**<<0.01**	**−0.149**	**<<0.01**	−0.097	0.008		
OAI	**0.167**	**<<0.01**	−0.105	0.004 *	−0.064	0.079		
ODI	**0.215**	**<<0.01**	−0.123	0.001 *	−0.054	0.138		
#Awakenings	−0.075	0.039	−0.027	0.461	−0.020	0.586		
WASO	−0.003	0.929	0.065	0.076	−0.022	0.538		
%N1	0.089	0.014	−0.071	0.052	−0.030	0.404		
%N2	−0.034	0.357	0.099	0.007 *	0.013	0.715		
%N3	0.034	0.355	−0.025	0.497	−0.044	0.23		
%REM	**−0.125**	**0.001**	−0.052	0.154	0.059	0.108		
TAI	**0.213**	**<<0.01**	**−0.158**	**<<0.01**	−0.115	0.002 *		
**BISP_Specific_ Features**								
**PSG Index**	***BW2_RP_Diag_***		***BW2_BE_1_***		***BWRes_B_min_***		***BWRes_BE_3_***	
	***ρ_S_***	***p*-Value**	***ρ_S_***	***p*-Value**	***ρ_S_***	***p*-Value**	***ρ_S_***	***p*-Value**
AHI	**0.308**	**<<0.01**	**−0.180**	**<<0.01**	0.054	0.136	0.045	0.214
OAHI	**0.261**	**<<0.01**	**−0.180**	**<<0.01**	0.098	0.007 *	0.028	0.435
OAI	**0.177**	**<<0.01**	**−0.173**	**<<0.01**	0.071	0.051	0.058	0.112
ODI	**0.247**	**<<0.01**	**−0.139**	**0.001**	0.019	0.61	0.072	0.047
#Awakenings	−0.033	0.372	−0.001	0.994	−0.006	0.876	0.035	0.331
WASO	0.071	0.05	0.078	0.031	−0.018	0.622	0.056	0.126
%N1	0.107	0.003 *	−0.061	0.093	0.023	0.527	0.028	0.441
%N2	−0.061	0.091	0.008	0.837	0.048	0.184	0.059	0.104
%N3	0.053	0.147	0.008	0.817	−0.075	0.04	−0.092	0.011
%REM	**−0.139**	**0.001**	0.048	0.192	−0.007	0.855	0.013	0.722
TAI	**0.225**	**<<0.01**	**−0.144**	**<<0.01**	0.068	0.062	0.068	0.06

PSG: polysomnographic; AHI: Apnea–Hypopnea Index; OAHI: Obstructive AHI; OAI: obstructive apnea index; ODI: oxygen desaturation index; #Awakenings: number of awakenings during total sleep time; WASO: wake after sleep onset; %N1: percentage of sleep spent in N1; %N2: percentage of sleep spent in N2; %N3: percentage of sleep spent in N3; %REM: percentage of sleep spent in REM; TAI: total arousal index. Those *p*-values below 10^−4^ appear as << 0.01. * Non-significant after Bonferroni correction. Statistically significant correlations (*p*-value < 0.01 after Bonferroni correction) appear in bold.

**Table 4:** Diagnostic performance achieved in the test set by each feature selected and each MLP optimized model for the binary classification in each severity threshold. Results are shown in terms of sensitivity (Se %), specificity (Sp %), accuracy (Acc %) and AUC.

**Threshold: AHI = 1 e/h**				
**Feature/model**	**Se**	**Sp**	**Acc**	**AUC**
*VLF_f2m*	44.5	72.3	53.9	0.605
*LF_BE_2_*	42.1	72.7	52.4	0.581
*HF_PE*	42.9	63.3	49.8	0.55
*BW2_RP_Diag_*	50.9	64.8	55.6	0.629
*BW2_BE_1_*	47.1	59.4	51.3	0.559
*BWRes_B_min_*	40.5	57.4	46.2	0.482
*BWRes_BE_3_*	41.5	57.4	46.9	0.513
*MLP1_Classic_*	52.3	59.4	54.7	0.6
*MLP1_Specific_*	76.3	38.3	**63.4**	**0.627**
**Threshold: AHI = 5 e/h**				
**Feature/Model**	**Se**	**Sp**	**Acc**	**AUC**
*VLF_f2m*	62.5	72.2	70.8	0.749
*LF_BE_2_*	56.3	74.4	71.7	0.67
*HF_PE*	45.5	72.1	68.2	0.628
*BW2_RP_Diag_*	60.7	77.7	75.2	0.747
*BW2_BE_1_*	56.3	70.1	68	0.671
*BWRes_B_min_*	58.9	45.3	47.3	0.567
*BWRes_BE_3_*	47.3	58.4	56.8	0.569
*MLP5_Classic_*	50.9	86.2	**81**	0.774
*MLP5_Specific_*	62.5	84.2	**81**	**0.791**
**Threshold: AHI = 10 e/h**				
**Feature/Model**	**Se**	**Sp**	**Acc**	**AUC**
*VLF_f2m*	63.8	76.7	75.6	0.784
*LF_BE_2_*	58	81.5	79.4	0.74
*HF_PE*	53.6	72.1	70.4	0.663
*BW2_RP_Diag_*	68.1	76	75.3	0.789

VLF: very low frequency; LF: low frequency; HF: high frequency; MLP: multi-layer perceptron; AHI: Apnea–Hypopnea Index. The highest ACC and AUC for each severity threshold are highlighted in bold.

**Figure A7:**
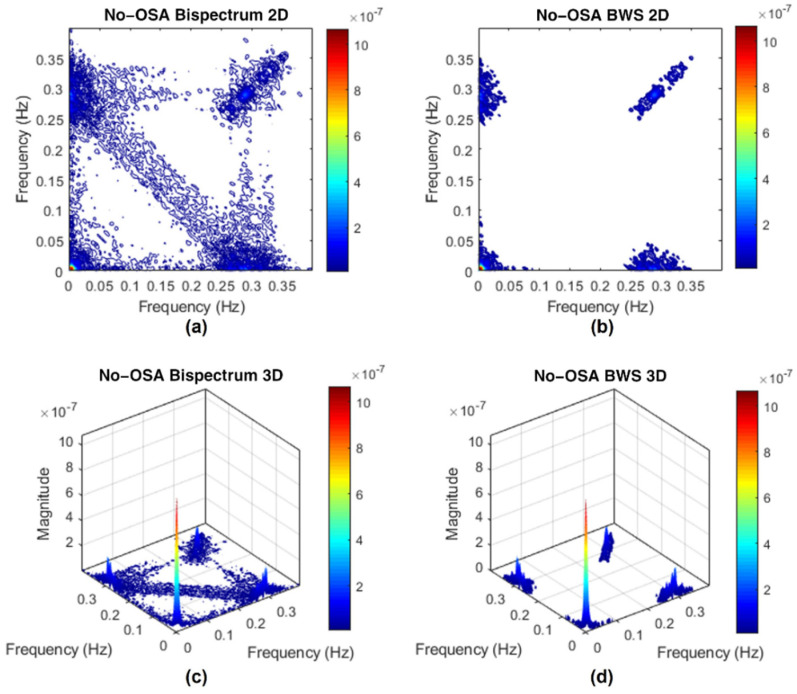
Bispectrum magnitude estimation with and without surrogate method for a no-OSA subject. (**a**) 2D bispectrum estimation; (**b**) 2D BWS estimation; (**c**) 3D bispectrum estimation; (**d**) 3D BWS estimation.

**Figure A8:**
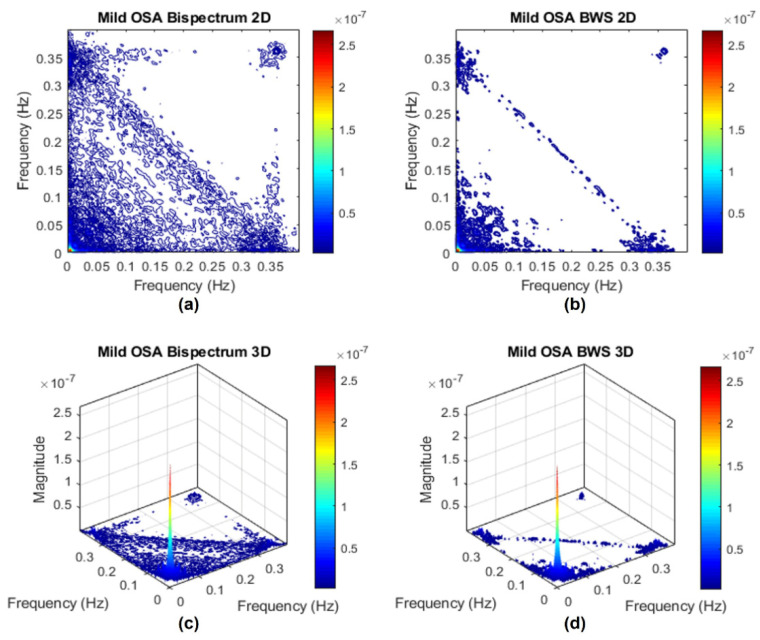
Bispectrum magnitude estimation with and without surrogate method for a mild OSA subject. (**a**) 2D bispectrum estimation; (**b**) 2D BWS estimation; (**c**) 3D bispectrum estimation; (**d**) 3D BWS estimation.

**Figure A9:**
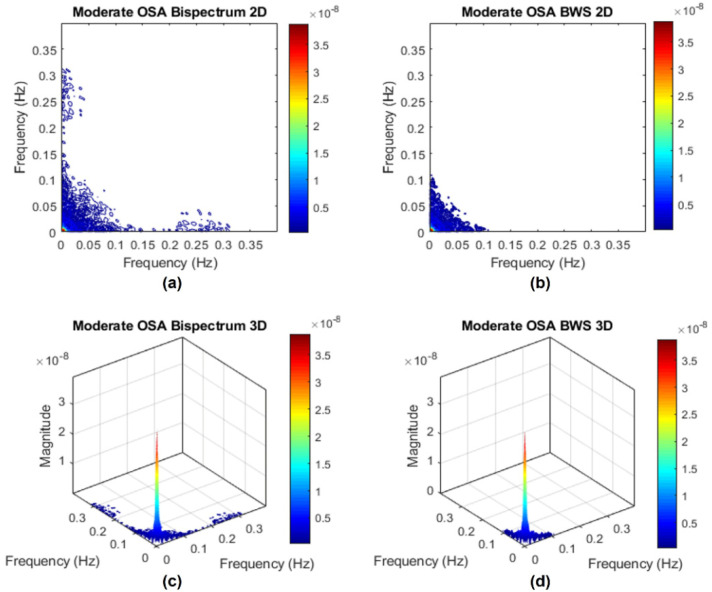
Bispectrum magnitude estimation with and without surrogate method for a moderate OSA subject. (**a**) 2D bispectrum estimation; (**b**) 2D BWS estimation; (**c**) 3D bispectrum estimation; (**d**) 3D BWS estimation.

**Figure A10:**
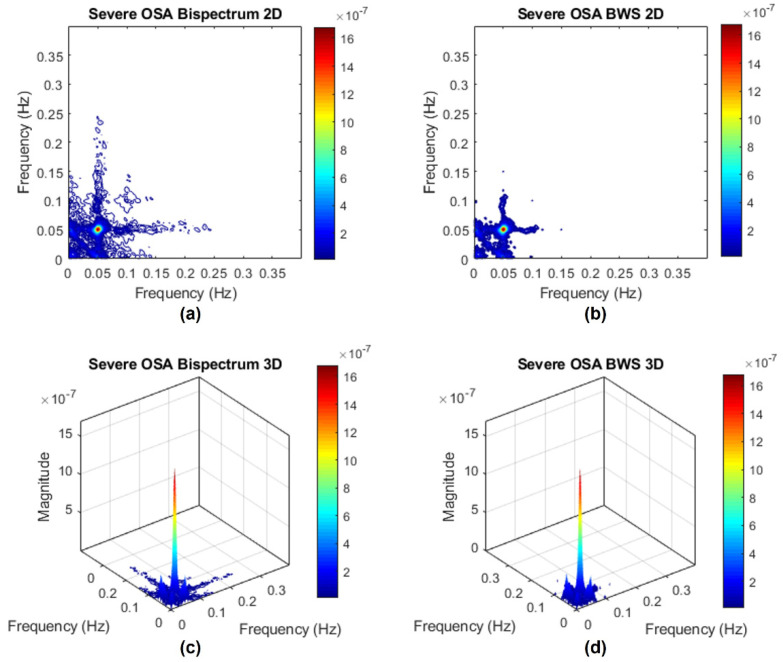
Bispectrum magnitude estimation with and without surrogate method for a severe OSA subject. (**a**) 2D bispectrum estimation; (**b**) 2D BWS estimation; (**c**) 3D bispectrum estimation; (**d**) 3D BWS estimation.

## Data Availability

The data presented in this study are available on request from the corresponding author. The data are not publicly available due to the privacy of individuals that participated in the study.

## Appendix A. Determination of HRV OSA-Specific Frequency Ranges and Averaged Bispectral Regions in the Training Set

The classic HRV spectral frequency ranges present some limitations when analyzing alterations in the autonomic nervous system as a result of pediatric OSA. This is the main motivation that led to us to the search of pediatric HRV OSA-specific frequency ranges in a recent work [27]. To perform the extraction of those spectral bands, we first established four severity groups based on the AHI. The HF frequency range, also known as the respiratory frequency band, is strongly modulated by age. For this reason, the search was separated in two different analyses to extract bands of interest based on the power spectral density. First, we performed an analysis over the 0–0.15 Hz frequency range, as it should not be altered by age [27]. The seek was performed through the evaluation of statistical differences (Mann–Whitney *U* test), frequency by frequency, between the amplitude of power spectral densities along this range for each severity group. Those frequency bands where, at least, two of the statistical tests showed statistically significant differences (*p*-value < 0.01 after Bonferroni correction) were selected as OSA-specific frequency ranges. This methodology allowed us to find two bands of interest, termed BW1 (0.001–0.005 Hz) and BW2 (0.028–0.074 Hz).

The second analysis performed was oriented to consider age modulation in the respiratory range. In this sense, age plays an important role in the location of the respiratory peak within HF (0.15–0.40 Hz) [59,60], meaning the assessment was an adaptive individual analysis. We first located the individual maximum amplitude inside the HF range. Therefore, as it was explained in the Discussion section in [27], a frequency range of 0.04 Hz around this respiratory peak was enough to assess pediatric OSA alterations in this adaptive individual frequency band, which we termed BWRes. A complete and detailed explanation of the whole methodology to extract those HRV OSA-specific frequency bands of interest can be found in the Methods section from our previous study [27].

Thus, now that the extraction of HRV OSA-specific frequency ranges has been described, below, we include the averaged bispectral distribution for each severity group, bounded by both classic and OSA-specific frequencies. Figure A1, Figure A2, Figure A3, Figure A4, Figure A5 and Figure A6 show the 3D representation for the VLF, LF, HF, BW1, BW2 and BWRes regions, respectively. The *x* and *y* axes from the BWRes region cannot be represented in terms of frequency, as it changes for each subject. That is why they appear in terms of the number of samples for this region.

## Appendix B. Diagnostic Performance of Bispectral Region Models with a Linear Discriminant Analysis Classifier

To perform the main automated classification analysis of this study, we optimized the MLP models for each severity threshold (results available in Table 4). However, for comparison purposes, we have included in this appendix the diagnostic performance achieved by each optimized feature subset following the same methodology as in our previous work [27]. To this effect, the diagnostic performance of the BISP_Classic_ and BISP_Specific_ feature subsets was evaluated through two models based on LDA (LDA_Classic_ and LDA_Specific_ models, respectively). Both classifiers were trained in the training set for each severity threshold, and the diagnostic performance was evaluated in the test set. Table A1 shows the results achieved by each model, along with the results from our previous work [27]. It can be observed that, as it is commented on in the Discussion section, the new diagnostic performance surpassed our previous classification results in terms of Acc and AUC in almost all the parameters. Only the individual Acc in 1 e/h for RP_BW1_ outperformed the LDA results presented here, but with a lower AUC.

**Table A1:** Diagnostic performance in the test set achieved following a spectral and a bispectral approach. The individual performances correspond to each relative power extracted in our previous work. Joint performance was assessed by constructing LDA classifiers.

	Feature/Model	AHI Threshold = 1 e/h				AHI Threshold = 5 e/h				AHI Threshold = 10 e/h			
		Se	Sp	Acc	AUC	Se	Sp	Acc	AUC	Se	Sp	Acc	AUC
	*RP*_VLF_	68.9	31.6	56.3	0.518	33.0	65.0	60.2	0.456	40.6	64.2	62.1	0.495
Previous work (frequency analysis approach)	*RP*_LF_	43.5	62.9	50.1	0.557	52.7	58.4	57.6	0.590	59.4	58.4	58.5	0.666
	*RP*_HF_	35.5	71.9	47.8	0.523	39.3	68.1	63.8	0.540	43.5	76.7	73.7	0.605
	*LF/HF*	37.7	70.3	48.7	0.540	45.5	66.8	63.7	0.567	49.3	70.8	68.8	0.643
	*RP*_BW1_	66.3	45.3	**59.2**	0.559	65.2	54.0	55.6	0.621	69.6	52.3	53.9	0.624
	*RP*_BW2_	32.7	78.1	48.1	0.591	45.5	82.0	76.6	0.670	58.0	78.2	76.4	0.751
	*RP_BWRes_*	45.5	56.6	49.3	0.532	44.6	64.0	61.2	0.571	49.3	64.0	62.6	0.628
	LDA Classic Bands	25.7	81.3	44.5	0.559	46.4	72.2	68.4	0.633	50.7	75.3	73.1	0.685
	LDA Bands of Interest	42.5	72.3	52.6	0.592	50.0	80.9	76.4	0.688	63.8	84.7	82.8	0.796
Present work (bispectral analysis approach)	LDA_Classic_	30.1	81.3	47.4	0.601	53.6	85.3	**80.6**	0.779	66.7	89.7	**87.6**	**0.847**
	LDA_Specific_	37.9	77.3	51.3	**0.615**	63.4	82.8	79.9	**0.792**	71.0	85.9	84.5	0.842

RP: relative power; VLF: very low frequency; LF: low frequency; HF: high frequency; LDA: linear discriminant analysis; AHI: Apnea–Hypopnea Index. The highest accuracy and AUC in each severity threshold are highlighted in bold.

## Appendix C. Surrogate Data Approach

### Appendix C.1. Testing for Nonlinearities

Despite the previous literature and evidence supporting the utility of nonlinear HRV analysis in the pediatric OSA context commented on in the Introduction section, testing the presence of nonlinearities in our database can increase the rationale in the selection of bispectral analysis over other traditional methods. To this effect, a surrogate data approach can be performed, which allows for these types of test [61]. The surrogate data generation techniques to test nonlinearities construct surrogate data from the original signal, preserving the power spectrum and data distribution while removing nonlinear properties [61,62]. After seeking for different surrogate data generation techniques, we decided to implement the iterative amplitude-adjusted Fourier transform method (IAAFT) [61,63], which is one of the most popular solutions [61].

Testing for nonlinearities with the IAAFT method first implies constructing a set of surrogate data. We performed this test over four randomly selected subjects from the training set, each one belonging to an OSA severity group (no-OSA: AHI < 1; mild OSA: 1 ≤ AHI < 5; moderate OSA: 5 ≤ AHI < 10; severe OSA: AHI ≥ 10). Thus, we generated 100 realizations of surrogate data from the original selected HRVs. The null hypothesis established was that the data represent a linear process, with all surrogate data being consistent with this hypothesis [61,63]. The next step was to compute a nonlinear measure for both original and all surrogate data and rank them in numerical order. We selected the Lempel-Ziv complexity (LZC) as a nonlinear measure that evaluates the generation rate of patterns present in a data sequence [64]. If the null hypothesis is true, the nonlinear measure evaluated in the original data should be indistinguishable from the measure computed for the surrogates [63]. However, for a two-sided test, if the nonlinear parameter is lower or higher than the rest of the measures computed in the surrogates, the null hypothesis can be rejected with a *p*-value < 0.02, and the existence of statistically significant nonlinearities in the original signal can be ensured. We confirmed that it occurs in the four HRV signals included, which means that the presence of nonlinearities in HRV signals under our study conditions has been demonstrated.

### Appendix C.2. Bispectrum with Surrogate

The surrogate data method can also be employed to check for the significance of bispectral peaks. Although peaks derived from frequency coupling alone should not appear in the bispectrum computation, sometimes they appear mixed with true peaks resulting from the frequency and phase coupling [62]. To solve this problem, the bispectrum with surrogate (BWS) computation method can be utilized [62]. Computing a bispectrum with and without the BWS technique and comparing these results allow assessing if the main peaks present in our study can be considered as significant.

The BWS technique is based on surrogate data. Thus, following the approach established in the subsection above, we performed BWS computation over the same four HRVs from the different OSA severity groups. Once the 100 realizations of surrogate data for each HRV were computed, we estimated the bispectrum for the original HRV and also for each surrogate. Then, over the surrogate set, we computed the mean and standard deviation of the bispectral magnitude, obtaining 100 means and standard deviations [62]. Based on these statistics, we constructed a 95% statistical threshold as the mean plus two times the standard deviation for each surrogate and selected the maximum threshold value as the threshold to apply over the original bispectrum estimation [62]. Any bispectral peak present in the original bispectrum estimation above the threshold computed was considered as significant, meaning BWS estimations were constructed canceling the bispectral magnitude values below the threshold. The next figures (Figure A7, Figure A8, Figure A9 and Figure A10) show the original bispectrum estimation along with the BWS estimation represented in 2D and 3D for each severity group.

The first outcome that can be appreciated is that the original bispectrum estimations (Subfigures a and c from each figure) present the main bispectral peak as well as some smaller peaks to a greater or lesser degree. After applying the BWS method (Subfigures b and d), most of these spurious peaks disappeared, with only the main peaks remaining, which are considered as significant [62]. In the Discussion section, we identified and discussed three phase coupling peaks: the VLF peak, the HF respiratory peak and a shift to the BW2 region range in the presence of OSA. For the no-OSA and mild OSA children (Figure A7 and Figure A8), the bispectral power is mainly concentrated around the VLF range, and a shift coupling is presented to a slight degree in a moderate OSA subject (Figure A9) and markedly in a severe OSA subject (Figure A10). For severe OSA, in fact, the main bispectral peak moved to the OSA-specific BW2 region. These results are in accordance with the conclusions extracted in the Discussion section. Another remaining coupling peak after BWS correction that deserves mention is the respiratory peak (typically inside the HF region). This peak can be appreciated in Figure A7 and Figure A8. However, it is absent in the moderate and severe OSA children, supporting our original conclusions about the coupling reduction around the respiratory peak and the redistribution of the bispectral power with OSA severity, especially in the severe group. Therefore, the results obtained through the BWS method support our original conclusions, increasing the robustness of our results.
