# Erratum: Martín-Montero et al. Bispectral Analysis of Heart Rate Variability to Characterize and Help Diagnose Pediatric Sleep Apnea. *Entropy* 2021, *23*, 1016

**DOI:** 10.3390/e23111375

**Published:** 2021-10-20

**Authors:** Adrián Martín-Montero, Gonzalo C. Gutiérrez-Tobal, David Gozal, Verónica Barroso-García, Daniel Álvarez, Félix del Campo, Leila Kheirandish-Gozal, Roberto Hornero

**Affiliations:** 1 Biomedical Engineering Group, University of Valladolid, 47002 Valladolid, Spain; gonzalo.gutierrez@gib.tel.uva.es (G.C.G.-T.); veronica.barroso@gib.tel.uva.es (V.B.-G.); dalvarezgo@saludcastillayleon.es (D.Á.); fsas@telefonica.net (F.d.C.); robhor@tel.uva.es (R.H.); 2 CIBER-BBN, Centro de Investigación Biomédica en Red en Bioingeniería, Biomateriales y Nanomedicina, 28029 Madrid, Spain; 3 Department of Child Health and the Child Health Research Institute, The University of Missouri School of Medicine, Columbia, MO 65212, USA; gozald@health.missouri.edu (D.G.); gozall@health.missouri.edu (L.K.-G.); 4 Sleep-Ventilation Unit, Pneumology Service, Río Hortega University Hospital, 47012 Valladolid, Spain

The author wishes to make the following correction to this paper [1]. Due to missing data, five rows were added to Table 4. Before:


**Threshold: AHI = 1 e/h**

**Feature/Model**

**Se**

**Sp**

**Acc**

**AUC**

***VLF_f2m***
44.572.353.90.605
***LF_BE_2_***
42.172.752.40.581
***HF_PE***
42.963.349.80.55
***BW2_RP_Diag_***
50.964.855.60.629
***BW2_BE_1_***
47.159.451.30.559
***BWRes_B_min_***
40.557.446.20.482
***BWRes_BE_3_***
41.557.446.90.513
***MLP1_Classic_***
52.359.454.70.6
***MLP1_Specific_***
76.338.3
**63.4**

**0.627**

**Threshold: AHI = 5 e/h**

**Feature/Model**

**Se**

**Sp**

**Acc**

**AUC**

***VLF_f2m***
62.572.270.80.749
***LF_BE_2_***
56.374.471.70.67
***HF_PE***
45.572.168.20.628
***BW2_RP_Diag_***
60.777.775.20.747
***BW2_BE_1_***
56.370.1680.671
***BWRes_B_min_***
58.945.347.30.567
***BWRes_BE_3_***
47.358.456.80.569
***MLP5_Classic_***
50.986.2
**81**
0.774
***MLP5_Specific_***
62.584.2
**81**

**0.791**

**Threshold: AHI = 10 e/h**

**Feature/Model**

**Se**

**Sp**

**Acc**

**AUC**

***VLF_f2m***
63.876.775.60.784
***LF_BE_2_***
5881.579.40.74
***HF_PE***
53.672.170.40.663
***BW2_RP_Diag_***
68.17675.30.789

After:


**Threshold: AHI = 1 e/h**

**Feature/Model**

**Se**

**Sp**

**Acc**

**AUC**

***VLF_f2m***
44.572.353.90.605
***LF_BE_2_***
42.172.752.40.581
***HF_PE***
42.963.349.80.550
***BW2_RP_Diag_***
50.964.855.60.629
***BW2_BE_1_***
47.159.451.30.559
***BWRes_B_min_***
40.557.446.20.482
***BWRes_BE_3_***
41.557.446.90.513
***MLP1_Classic_***
52.359.454.70.600
***MLP1_Specific_***
76.338.3
**63.4**

**0.627**

**Threshold: AHI = 5 e/h**

**Feature/Model**

**Se**

**Sp**

**Acc**

**AUC**

***VLF_f2m***
62.572.270.80.749
***LF_BE_2_***
56.374.471.70.670
***HF_PE***
45.572.168.20.628
***BW2_RP_Diag_***
60.777.775.20.747
***BW2_BE_1_***
56.370.168.00.671
***BWRes_B_min_***
58.945.347.30.567
***BWRes_BE_3_***
47.358.456.80.569
***MLP5_Classic_***
50.986.2
**81.0**
0.774
***MLP5_Specific_***
62.584.2
**81.0**

**0.791**

**Threshold: AHI = 10 e/h**

**Feature/Model**

**Se**

**Sp**

**Acc**

**AUC**

***VLF_f2m***
63.876.775.60.784
***LF_BE_2_***
58.081.579.40.740
***HF_PE***
53.672.170.40.663
***BW2_RP_Diag_***
68.176.075.30.789
***BW2_BE_1_***
47.876.073.40.692
***BWRes_B_min_***
56.550.651.10.557
***BWRes_BE_3_***
55.159.459.00.614
***MLP10_Classic_***
43.596.5
**91.7**

**0.847**

***MLP10_Specific_***
66.791.689.30.841

The *Entropy* Editorial Office would like to apologize for any inconvenience caused to readers by these changes. The changes do not affect the scientific results. The published version will be updated on the article webpage with a reference to this Erratum.
